# Extra-Virgin Olive Oil Phenolics in IBD-Associated Vascular Risk

**DOI:** 10.3390/molecules31111827

**Published:** 2026-05-26

**Authors:** Roko Šantić, Marko Kumrić, Lovre Martinović, Marino Vilović, Iris Jerončić Tomić, Ivan Cvitković, Joško Božić

**Affiliations:** 1 Department of Pathophysiology, University of Split School of Medicine, 21000 Split, Croatia; roko.santic@mefst.hr (R.Š.); marko.kumric@mefst.hr (M.K.); lovre.martinovic@mefst.hr (L.M.); marino.vilovic@mefst.hr (M.V.); 2 Laboratory for Cardiometabolic Research, University of Split School of Medicine, 21000 Split, Croatia; 3 Department of Public Health, University of Split School of Medicine, 21000 Split, Croatia; iris.jeroncic@mefst.hr; 4 Department of Anesthesiology and Intensive Care, University Hospital of Split, Spinciceva 1, 21000 Split, Croatia; ivcvitkovic@kbsplit.hr

**Keywords:** extra-virgin olive oil, oleocanthal, oleacein, hydroxytyrosol, secoiridoids, inflammatory bowel disease, endothelial dysfunction, platelet activation, gut–vascular axis, thromboinflammation

## Abstract

High-phenolic extra-virgin olive oil (EVOO) is a chemically dynamic bioactive matrix in which cultivar, ripening stage, processing, storage, and digestion shape the final profile of phenolic alcohols and secoiridoids. In inflammatory bowel disease (IBD), chronic intestinal inflammation is associated with barrier dysfunction, dysbiosis, systemic immune activation, endothelial injury, platelet hyperreactivity, and increased cardiovascular risk. This narrative review evaluates whether EVOO phenolics may intersect the gut–endothelium–platelet axis linking IBD to vascular and thromboinflammatory complications. The review focuses on hydroxytyrosol, tyrosol, oleuropein- and ligstroside-derived secoiridoids, oleocanthal, and oleacein, with emphasis on their biosynthetic origin, processing-driven transformations, bioavailability, metabolism, and biological targets. Current evidence supports plausible effects on epithelial barrier integrity, TLR4/NF-κB signalling, Nrf2-mediated antioxidant defence, oxidised LDL formation, endothelial activation, and platelet-related pathways. Nevertheless, direct clinical evidence in IBD patients remains limited, and most cardiovascular-relevant findings are extrapolated from non-IBD human trials, animal studies, or in vitro models. Chemically characterised, biomarker-anchored intervention trials are needed before high-phenolic EVOO can be considered a validated strategy for modifying cardiovascular risk in IBD.

## 1. Introduction

Patients with inflammatory bowel disease (IBD)—Crohn’s disease and ulcerative colitis—carry a measurably elevated burden of cardiovascular events, including acute coronary syndrome, stroke, heart failure, and venous thromboembolism, beyond what traditional risk factors predict [1,2,3,4,5,6]. Mechanistic interrogation of this excess risk has converged on a gut–endothelium–platelet continuum: barrier dysfunction and dysbiosis sustain low-grade endotoxaemia, which drives endothelial activation, lipoprotein oxidation, and prothrombotic platelet phenotypes [6,7,8,9]. The translational question is whether dietary bioactives that act on these molecular nodes could plausibly modify cardiovascular risk in IBD. Olive oil is frequently invoked in this discussion, yet the literature suffers from a persistent imprecision: the term “olive oil” is used without distinguishing between extra-virgin grades dense in phenolic secoiridoids and refined oils stripped of those compounds during industrial processing [10,11,12,13,14,15].

This review addresses that imprecision by treating high-phenolic EVOO as a chemically defined exposure. EVOO is not synonymous with “olive oil” in any biomedically meaningful sense. EVOO is a chemically defined bioactive matrix composed mainly of a triacylglycerol fraction, approximately 98% by mass and dominated by oleic acid, and a minor fraction containing tocopherols, squalene, triterpenes, and hydrophilic phenolic compounds. EVOO is a chemically defined bioactive matrix composed mainly of a triacylglycerol fraction, approximately 98% by mass and dominated by oleic acid, and a minor fraction containing tocopherols, squalene, triterpenes, and hydrophilic phenolic compounds. In this review, high-phenolic EVOO is operationally defined as EVOO providing at least 5 mg of hydroxytyrosol and its derivatives per 20 g of oil, corresponding to approximately ≥250 mg/kg of these compounds; by contrast, refined olive oils may contain <50 mg/kg phenolics because these compounds are largely removed during refining [10,11,16,17,18]. Within that minor fraction, secoiridoids (oleuropein- and ligstroside-derived aglycones, including the dialdehydic forms oleocanthal and oleacein) and phenolic alcohols (hydroxytyrosol and tyrosol) account for the bulk of the bioactivity for which the EU regulator has granted a health claim [16]. Refined olive oil, having been deodorised and decoloured, retains the lipid backbone but is largely depleted of these phenolics and accordingly lacks the antioxidant and anti-inflammatory activities tied to them [11,14].

The present narrative review therefore takes the minor phenolic fraction of high-phenolic EVOO as its principal subject and the IBD-associated gut–endothelium–platelet axis as its clinical context. The aim is not to defend a generic Mediterranean dietary pattern, nor to argue that EVOO prevents cardiovascular events in IBD—an evidential claim the literature does not support—but to ask, with the precision the journal *Molecules* expects, whether the molecular profile of high-phenolic EVOO maps onto the pathogenic pathways most relevant to IBD-associated cardiovascular complications. Consistent with the journal’s chemistry-forward tradition, the review is organised around compounds, transformations, and mechanisms rather than around food pattern epidemiology.

The novelty of this review does not lie in re-stating that IBD carries increased cardiovascular risk, nor in re-summarising the broad literature on olive oil and intestinal inflammation, both of which are already well covered [3,6,19,20]. Rather, this review (i) treats high-phenolic EVOO as a chemically defined exposure rather than a food category, (ii) focuses on specific molecules—oleocanthal, oleacein, hydroxytyrosol, tyrosol, and oleuropein- and ligstroside-derived aglycones—and on the in vivo metabolites that actually circulate after ingestion, and (iii) critically examines whether those molecules and metabolites, at exposures realistically achievable from native EVOO consumption, intersect the pathogenic nodes that link IBD with endothelial dysfunction, lipoprotein oxidation, and arterial and venous thromboinflammation. Throughout, mechanistic enthusiasm is benchmarked against pharmacokinetic realism and against the fidelity of each cited exposure to native EVOO.

## 2. Methods and Search Strategy

This narrative review was prepared in accordance with SANRA recommendations for transparent reporting of non-systematic reviews. PubMed/MEDLINE, Scopus, and Web of Science were searched between 2 and 25 April 2026, supplemented by hand-searching, targeted Google Scholar queries, and searches of the EFSA Journal and the EU Register of authorised health claims. Search terms combined three domains: EVOO exposure and bioactive compounds, including high-phenolic EVOO, oleocanthal, oleacein, oleuropein, ligstroside, hydroxytyrosol, tyrosol, and olive secoiridoids; mechanistic targets, including endothelial function, oxidised LDL, platelet activation, TLR4/NF-κB, Nrf2, intestinal barrier function, gut microbiota, endotoxaemia, and NETosis; and clinical context, including IBD, ulcerative colitis, Crohn’s disease, cardiovascular disease, atherosclerosis, and venous thromboembolism.

Eligible records addressed EVOO chemistry, processing, bioavailability, metabolism, mechanistic effects of defined olive oil compounds or chemically characterised EVOO, cardiovascular outcomes, or IBD-associated cardiovascular risk. Human, animal, and in vitro studies were included, but evidence was interpreted hierarchically. Studies without adequate oil characterisation, studies conflating extra-virgin and refined olive oil, and Mediterranean diet studies without an isolable EVOO component were not used to support molecule-specific mechanistic claims. References were checked against original sources or bibliographic records whenever possible.

## 3. Chemical Identity, Formation, and Bioavailability of High-Phenolic EVOO

### 3.1. Chemical Architecture

EVOO is, by EU and IOC definition, a virgin olive oil obtained exclusively by mechanical means with free acidity ≤ 0.8% and meeting strict sensory criteria [10]. Its chemistry can be partitioned into a saponifiable lipid fraction (~98%, almost entirely triacylglycerols with oleic acid as the dominant fatty acid, 55–83 mol%) and an unsaponifiable plus minor polar fraction (~2%) housing the compounds with disproportionate biological activity [10,11].

Among the simple phenols, hydroxytyrosol (3,4-dihydroxyphenylethanol) and tyrosol (4-hydroxyphenylethanol) are the two principal phenolic alcohols in EVOO. Free hydroxytyrosol concentrations in fresh EVOO typically range from 1 to 10 mg/kg and rise to 10–50 mg/kg upon storage as secoiridoids hydrolyse; tyrosol follows analogous kinetics [11,21]. Both arise primarily from hydrolysis of secoiridoid glucosides during fruit processing and storage and are also produced by gut and hepatic metabolism after ingestion. The catechol (hydroxytyrosol) and phenol (tyrosol) functionalities underpin direct radical scavenging—hydroxytyrosol’s catechol motif yields particularly favourable hydrogen atom and single-electron transfer kinetics—but their biological relevance extends well beyond classical antioxidant chemistry to Nrf2 induction, NF-κB suppression, and modulation of intestinal and endothelial barrier proteins [22,23,24]. The fact that the EU health claim under Regulation 432/2012 is anchored on hydroxytyrosol and its derivatives (≥5 mg per 20 g of olive oil) reflects the regulatory recognition that this specific chemical class, rather than olive oil per se, carries the LDL-protective signal [16,17].

The secoiridoid fraction is more specific to *Olea europaea* and is the main chemical reason why high-phenolic EVOO differs from refined olive oil. Oleuropein and ligstroside, abundant in the fruit but largely absent from the final oil, are oleoside-type secoiridoid glycosides in which the oleoside-11-methyl-ester core is conjugated, via the C-7 carboxyl, to hydroxytyrosol or tyrosol, respectively [25,26]. The defining structural element is the oleosidic moiety: a methylated open-ring iridoid with an enol ether vinyl group and an exocyclic methyl ester, distinct from the secologanin scaffold of most other secoiridoid-producing plants and produced by an idiosyncratic CYP72-driven oxidative ring-opening reaction unique to *Olea* [27]. During oil extraction, glucosidase- and esterase-driven hydrolysis cleaves the glucose moiety, liberating the aglycones, which subsequently undergo decarboxymethylation to yield a family of dialdehydic species. Two of these dominate the phenolic profile of fresh, well-made EVOO and account for much of its sensory pungency and pharmacological activity: oleocanthal, the dialdehydic decarboxymethyl form of ligstroside aglycone, structurally a tyrosol ester of an open dialdehyde elenolic-acid fragment, and oleacein, the analogous dialdehydic decarboxymethyl form of oleuropein aglycone, bearing a hydroxytyrosol moiety in place of tyrosol [26,28,29,30]. Oleocanthal concentrations in commercial high-phenolic EVOOs typically range from 100 to 600 mg/kg, with monovarietal premium oils reaching 800 mg/kg; oleacein follows a similar distribution [28,31]. Both compounds carry two reactive aldehyde groups—an α,β-unsaturated aldehyde and an aliphatic aldehyde—whose electrophilicity underlies both their pharmacology and their pharmacokinetic intractability (Section 4).

Within this fraction, oleocanthal and oleacein are particularly relevant to the cardiovascular argument developed in this review. Oleocanthal was identified as a non-steroidal anti-inflammatory entity with cyclooxygenase-1 (COX-1) and COX-2 inhibition profiles approximating those of ibuprofen on a molar basis [29,30]. Oleacein, structurally analogous but bearing a catechol moiety inherited from hydroxytyrosol, exhibits potent NF-κB and TLR4 pathway suppression in macrophages and adipocytes and direct vascular effects [32,33,34].

Other minor constituents are relevant but less central to the present argument. α-tocopherol (typically 100–300 mg/kg) contributes lipid phase antioxidant activity, particularly in protecting unsaturated fatty acids from peroxidation; squalene (200–8000 mg/kg) provides additional lipid phase reductive capacity [10]. Pentacyclic triterpenes such as maslinic and oleanolic acids, although biologically active, are present at relatively low concentrations and are not central to the present argument; they are mentioned where directly relevant.

By contrast, oleic acid provides the lipid background rather than the distinctive high-phenolic signal. As the dominant fatty acid, it may contribute to favourable cholesterol partitioning, modest improvements in insulin sensitivity, and lower oxidative susceptibility relative to polyunsaturated fats. However, refined olive oil is also oleic-acid-rich and phenolic-poor. The biomarker differences between refined and high-phenolic oils therefore point mainly to the phenolic fraction, not to fatty acid composition alone [35,36,37]. The main phenolic constituents discussed in this section are summarised in Table 1 according to their chemical origin, principal metabolic fate, and proposed relevance to IBD-associated vascular risk.

Together, these compounds indicate that the distinctive bioactivity of high-phenolic EVOO is driven mainly by its secoiridoid-derived phenolic fraction, rather than by the oleic-acid backbone shared with refined olive oil.

### 3.2. From Olive Fruit to Bottle: Biosynthesis and Processing-Driven Transformations

The biological relevance of EVOO phenolics depends not only on their chemical identity, but also on how they are formed, transformed during extraction, and degraded during storage or heating.

In the olive fruit, oleuropein and ligstroside are end products of a secoiridoid biosynthetic pathway shared, in part, with the monoterpenoid indole alkaloid pathway of *Catharanthus roseus*. The sequence proceeds from geraniol through 8-oxogeranial to nepetalactol via iridoid synthase (OeISY) [44], and onwards to 7-deoxyloganic acid, 7-epi-loganic acid, and 7-epi-loganin. A pair of bi-functional CYP72 cytochromes (OeOMES, OeSXS) catalyses the unusual oxidative C–C bond cleavage that opens the iridoid ring to yield oleoside methyl ester, the unique secoxyiridoid scaffold of the olive [27]. The oleoside is then esterified to hydroxytyrosol or tyrosol to give oleuropein or ligstroside. A fruit β-glucosidase (OeGLU) governs flux through the pathway in planta, and its silencing collapses the entire secoiridoid pool, demonstrating that the network is enzymatically rather than thermodynamically determined [44,45].

Extraction does not simply transfer fruit phenolics into oil; it actively reshapes them. When olives are crushed, compartmental separation between substrate and enzyme breaks down. Endogenous β-glucosidase, esterase, peroxidase, and polyphenol oxidase act in concert—and in competition—to deglycosylate oleuropein and ligstroside, generating the corresponding aglycones, and to demethyl-decarboxylate them to the dialdehydic species [25,28,46,47]. The dialdehydic aglycones of ligstroside and oleuropein are, respectively, oleocanthal and oleacein. Two olive methylesterases (OeEAME1, OeEAME2) accelerate this conversion [48]. Malaxation conditions—time and temperature—are decisive: extended malaxation at 30–37 °C maximises β-glucosidase output and shifts the phenolic profile toward oleocanthal and oleacein, while concurrently reducing total phenolic mass through oxidative loss [28,31]. Extraction technology (two- vs. three-phase decanters), seed crushing, and water addition all modulate the final profile [46,47].

The scale of this process depends strongly on cultivar, ripening stage, and agronomy. Cultivar is the primary determinant of phenolic potential, reflecting genetic differences in the expression of OeISY, OeGLU, OeEAME methylesterases, and the CYP72 ring-opening cytochromes [25,27,44,45,48]. Comparative transcriptomic profiling between high- and low-phenol cultivars at fruit developmental stages identifies coordinated upregulation of glucosyltransferase, deoxyxylulose-5-phosphate reductoisomerase, geraniol synthase, and secologanin-synthase-like genes as the principal drivers of secoiridoid accumulation [44]. Ripening shifts the spectrum: green olives are typically richer in oleuropein and ligstroside derivatives, whereas advanced maturation favours simpler phenolic alcohols and a lower total content as polyphenol oxidase and peroxidase activities rise [31]. Irrigation regime, altitude, soil, and harvest timing further modify yields, with mild water stress and earlier harvest typically associated with higher phenolic concentrations.

The phenolic profile continues to change after bottling. Phenolic content declines pseudo-first-order over storage at rates strongly dependent on temperature, light, and headspace oxygen [21,49]. Cold, dark storage in inert headspace can preserve secoiridoid content for 12–18 months, while ambient or elevated storage degrades oleocanthal and oleacein within months [28,49]. Heating EVOO further compromises bioactivity: domestic frying temperatures (180–240 °C) reduce oleocanthal-associated COX-inhibitory activity by up to a third even when chromatographic concentrations decline less, indicating partial structural modification of the dialdehydic motif [14,50].

The implication for biomedical research is straightforward: two oils legally labelled “extra-virgin” can differ tenfold or more in oleocanthal, oleacein, and total hydroxytyrosol equivalents. Any clinical or mechanistic claim that is not anchored to a chemically characterised oil is, at best, uninterpretable [17,18].

### 3.3. Bioaccessibility, Bioavailability, Metabolism, and the Matrix Effect

After ingestion, hydroxytyrosol and tyrosol are absorbed in the small intestine in a dose-dependent manner; peak plasma concentrations occur within 30–60 min, but circulating free hydroxytyrosol is scarce because >95% is rapidly conjugated by intestinal and hepatic phase II enzymes—principally sulfotransferases and UDP-glucuronosyltransferases-to hydroxytyrosol-3-O-sulfate, hydroxytyrosol-glucuronides, and methylated derivatives such as homovanillic alcohol [51,52,53,54,55,56,57,58,59,60]. Tyrosol shows analogous behaviour, with the additional complication that endogenous tyrosol arises from dopamine metabolism, complicating biomarker interpretation in non-controlled diets [53,54,61]. Phase II metabolites have, in some assays, attenuated direct antioxidant capacity relative to parent compounds [62], yet retain Nrf2-inducing and NF-κB-suppressing activities at target tissues, where deconjugation by tissue sulfatases and β-glucuronidases can liberate active aglycones [22,38].

Secoiridoids behave less like stable circulating parent compounds and more like precursors of several downstream metabolites. Oleuropein and ligstroside aglycones are partially absorbed but largely hydrolysed in the gastrointestinal tract to release hydroxytyrosol and tyrosol, which then follow the phase II routes described above. Colonic microbiota convert residual secoiridoids and phenolic alcohols to phenolic acid metabolites such as 3,4-dihydroxyphenylacetic acid, 3-hydroxyphenylpropionic acid, and homovanillic acid, some of which retain biological activity [63,64,65,66].

Oleocanthal and oleacein are especially difficult to follow pharmacokinetically because their dialdehyde groups react spontaneously with primary amines in biological fluids—glycine, lysine residues on proteins, and free amino acids—forming Schiff bases within minutes [67,68]. Direct plasma quantification of intact oleocanthal therefore systematically underestimates exposure. The Darakjian et al. glycine adduct strategy, which derivatises oleocanthal to a stable diagnostic adduct, provided the first reliable in vivo pharmacokinetic data [67]. In situ rat intestinal perfusion shows oleocanthal absorption with extensive intestinal first-pass metabolism, generating hydrated, hydrogenated, and hydroxylated phase I products and their phase II glucuronides; oleacein behaves similarly, with intestinal permeability comparable to naproxen [28,69,70,71]. Tissue distribution data demonstrate that oleocanthal and its metabolites reach liver, kidney, adipose tissue, and brain [69], with brain penetrance of olive oil phenolics specifically reviewed by Rodríguez-Morató and colleagues in the context of neurodegenerative biology [72]. Inter-individual variability in hydroxytyrosol bioavailability—driven by polymorphisms in conjugating enzymes, microbiome composition, and matrix factors—has been systematically reviewed and is increasingly recognised as a determinant of clinical response to phenolic interventions [73].

Delivery in an oil matrix matters. Hydroxytyrosol bioavailability is meaningfully higher when delivered in an oil matrix than in an aqueous vehicle, plausibly because lipid coingestion modulates gastric emptying, micellar partitioning, and first-pass glucuronidation [55,56,58]. EVOO is therefore not a passive carrier; the matrix alters absorption kinetics and metabolite profile. Functional emulsion approaches that re-incorporate hydroxytyrosol or oleuropein into oily matrices preserve high in vitro bioaccessibility (>80% at the gastric–intestinal interface), supporting the principle that the food matrix is part of the active formulation [74].

The composite picture is that the bioactive molecules circulating in vivo after EVOO ingestion are predominantly conjugated phenolic alcohols, glycine-adducted secoiridoid derivatives, microbial phenolic acid metabolites, and tissue-delivered free aglycones following local deconjugation. Mechanistic claims about pathways such as endothelial NF-κB or platelet COX must be evaluated against this metabolic landscape, not against the parent compounds in the oil. Quantitatively, the gap between in vitro mechanistic concentrations (frequently low- to high-micromolar) and in vivo postprandial plasma concentrations is substantial: a controlled human crossover study delivering 5 mg of hydroxytyrosol in an EVOO matrix produced a peak plasma free hydroxytyrosol concentration of only ~3.8 ng/mL (~25 nM) at 30 min [56], and a recent comprehensive review of hydroxytyrosol bioavailability emphasises that virtually all in vivo activity must be reconciled with conjugated metabolites circulating at mid-nanomolar to low micromolar concentrations rather than with parent aglycones at the supraphysiological exposures used in many cell systems [73]. Mechanistic enthusiasm in subsequent sections is benchmarked against this exposure realism.

## 4. IBD as a Systemic Inflammatory Disease with Cardiovascular Consequences

IBD is no longer regarded as a purely luminal disease. Population-scale data establish that Crohn’s disease and ulcerative colitis confer excess risks of myocardial infarction, ischaemic stroke, heart failure, venous thromboembolism, and cardiovascular mortality, with hazard ratios typically in the range of 1.2–1.7 for arterial events and substantially higher during disease flares and hospitalisation [1,2,3,4,5,6]. A 2025 meta-analysis encompassing 2.2 million individuals demonstrated elevated incident cardiovascular events across both phenotypes, with measurable modulation by therapy class [1]. Diet has emerged as a candidate modifier of IBD onset and course, with systematic review and meta-analytic evidence linking dietary patterns rich in plant bioactives to reduced incident IBD risk [75], and a paediatric randomised trial showing that adherence to a Mediterranean-style diet improves clinical and inflammatory indices in active IBD [76]; mechanistic and translational reviews specifically on olive oil components in IBD have catalogued multiple plausible intersection points between EVOO bioactives and intestinal inflammation [20]. Meta-analyses confirm raised acute coronary syndrome and stroke risk, with Crohn’s disease carrying a particularly pronounced cerebrovascular signal [2,3,4]. Heart failure incidence is also raised, plausibly through chronic cytokine-mediated cardiac remodelling [5].

Beyond hard endpoints, IBD is characterised by surrogate vascular phenotypes that anticipate clinical disease: brachial flow-mediated dilation is reduced even in paediatric IBD prior to traditional risk factor accumulation [77,78]; pulse wave velocity and augmentation index are elevated and track inflammatory activity, leading expert panels to designate aortic stiffening as an extraintestinal manifestation of IBD [79,80,81,82,83]. Effective anti-inflammatory therapy partially restores endothelial function and glycocalyx integrity, indicating that the vascular phenotype is dynamically inflammation-driven rather than fixed [79].

The mechanistic substrate underlying these observations is a chronic systemic inflammatory state characterised by elevated CRP, IL-6, TNF-α, and IL-1β, persistent neutrophil and platelet activation, and lipid peroxidation [84,85,86,87,88]. This state, rather than any single classical risk factor, appears to mediate the IBD-specific vascular signature. Importantly, the magnitude of the vascular signal in IBD is not fully accounted for by traditional Framingham-type risk factors: IBD cohorts have, on average, lower body mass index and similar or only modestly altered lipid profiles relative to matched controls, yet exhibit higher rates of arterial and venous events [1,2,3,4,89]. The implication is that interventions targeting traditional risk factors alone—statins and antihypertensives—are unlikely to fully neutralise IBD-specific cardiovascular risk, opening the conceptual space for adjunctive interventions aimed specifically at the inflammation–barrier–thromboinflammation axis. Therapy class also matters: nationwide and meta-analytic data indicate that anti-TNF biologics and JAK inhibitors modify cardiovascular risk in directions that are still being mapped, with anti-TNF therapy associated with reductions in vascular inflammation surrogates and JAK inhibition flagged for thromboembolic signal in older or higher risk subsets [1,5,89]. Any nutritional intervention proposed for IBD must therefore be evaluated within, not in isolation from, this evolving therapeutic landscape.

## 5. The Gut–Endothelium–Platelet Axis: Conceptual Core of the Argument

### 5.1. Barrier Dysfunction and Dysbiosis

Active IBD disrupts tight junction architecture (claudins, occludin, ZO-1) and the mucin layer, increasing paracellular permeability [90,91]. Dysbiosis—loss of *Faecalibacterium prausnitzii* and other short-chain fatty acid producers and the expansion of mucosa-adherent *Enterobacteriaceae*—reduces luminal SCFA generation and alters bile acid metabolism, weakening epithelial repair [9,90,91]. Bacterial endotoxin (lipopolysaccharide, LPS) and microbial-associated molecular patterns translocate to the portal and systemic circulations, producing a state of low-grade endotoxaemia [7]. The wider gut microbiota–cardiovascular literature provides additional context for how this translocation drives systemic vascular inflammation through metabolic and immune intermediates [92], and EVOO has been specifically reviewed in the gut–brain–cardiometabolic axis as a microbiota- and mucosal-immunity-modulating food matrix [19].

### 5.2. TLR4–NF-κB Activation and Oxidative Stress

Circulating LPS engages TLR4 on monocytes, macrophages, and endothelial cells, triggering NF-κB-driven transcription of TNF-α, IL-6, IL-1β, MCP-1, ICAM-1, and VCAM-1 [92]. Concurrently, mitochondrial and NADPH-oxidase-derived reactive oxygen species deplete glutathione and oxidise LDL and overwhelm Nrf2-driven antioxidant defences [84,85,86].

### 5.3. Endothelial Activation

Persistent cytokine and oxidative stimuli reduce nitric oxide bioavailability through eNOS uncoupling and tetrahydrobiopterin depletion, upregulate adhesion molecules, and destabilise the endothelial glycocalyx, producing the FMD impairment and PWV elevation observed in IBD cohorts [77,78,79,80,81,82,83,93,94].

### 5.4. Platelet Activation and Immunothrombosis

IBD platelets are hyperreactive, with elevated P-selectin externalisation, increased platelet–leukocyte aggregate formation, and altered mean platelet volume tracking disease activity [95,96,97,98,99,100,101]. Chronic endotoxaemia and cytokine signalling prime platelet COX-1, raising thromboxane A2 generation; platelets in turn release prothrombotic and pro-inflammatory mediators (CD40L, RANTES, sphingosine-1-phosphate). Neutrophil extracellular traps (NETs), formed in IBD mucosa and in the systemic circulation, scaffold immunothrombosis: NET-bound histones and tissue factor activate platelets and the coagulation cascade, producing a thrombotic microenvironment that links luminal neutrophil activation to systemic venous and arterial thrombotic risk [102,103,104,105,106].

This axis is used here as a conceptual framework for organising mechanistic and translational evidence, rather than as a clinically validated EVOO-specific pathway in IBD. Although several EVOO-derived compounds have documented activity on barrier integrity, TLR4–NF-κB signalling, oxidative tone, endothelial activation, platelet COX, or NET-related pathways, most of these links are currently supported by preclinical, ex vivo, or non-IBD human evidence rather than by direct vascular or platelet intervention trials in IBD patients. 

**Figure 1:**
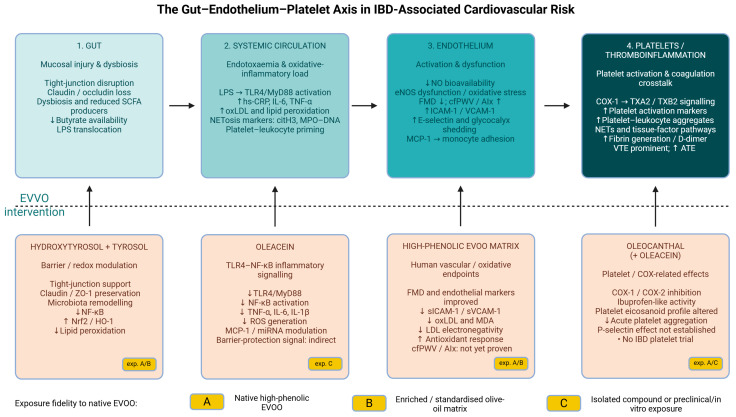
The gut–endothelium–platelet axis in IBD-associated cardiovascular risk and proposed high-phenolic EVOO intervention points. The (**upper row**) summarises the proposed pathogenic cascade from mucosal injury, dysbiosis, and barrier dysfunction to systemic endotoxaemia, oxidative inflammatory load, endothelial activation, platelet hyperreactivity, NET-associated thromboinflammation, and arterial or venous cardiovascular phenotypes. The (**lower row**) maps major EVOO-derived bioactives to mechanistically plausible intervention points along this axis. Exposure fidelity categories distinguish evidence derived from native high-phenolic EVOO, enriched or standardised olive oil matrices, and isolated compounds or preclinical/in vitro exposures. The scheme is hypothesis-generating and should not be interpreted as proof that EVOO prevents cardiovascular events in IBD. Created in https://BioRender.com.

This conceptual framework is summarised in Figure 1, which separates the IBD-driven pathogenic cascade from the proposed EVOO intervention points and distinguishes native high-phenolic EVOO evidence from enriched-matrix and isolated-compound data.

## 6. Component-by-Component Mechanistic Relevance

### 6.1. Hydroxytyrosol and Tyrosol

Hydroxytyrosol is the most extensively characterised EVOO phenolic alcohol. In intestinal epithelial cell models challenged with oxysterols or LPS, hydroxytyrosol and oleuropein aglycone reduce intracellular ROS, attenuate NF-κB activation, and suppress IL-8 secretion [107]. Crucially, the major circulating phase II metabolites—hydroxytyrosol-glucuronide and hydroxytyrosol-sulfate—preserve barrier protective activity in HUVEC monolayers under LPS stimulation, with reduced ICAM-1 expression and attenuated paracellular permeability, addressing the long-standing concern that conjugation eliminates bioactivity [38]. Hydroxytyrosol activates Nrf2/ARE-driven phase II antioxidant enzymes (HO-1, NQO1, GCLC) and modulates autophagy, providing redox and proteostatic resilience to inflamed epithelial and endothelial cells [23,24,108]. In dextran sulfate sodium (DSS) colitis, hydroxytyrosol restores ZO-1 and occludin expression, reduces colonic TNF-α, IL-6, and IL-1β, and reshapes the microbiota toward *Lactobacillus* and *Bifidobacterium*, providing a coherent multi-mechanistic preclinical signal of relevance to IBD [39]. Tyrosol shares some antioxidant and NF-κB-modulating activities but with lower potency, consistent with the absence of its catechol moiety.

### 6.2. Oleuropein- and Ligstroside-Derived Secoiridoids

Oleuropein aglycone and its dialdehydic congeners are the immediate precursors of oleocanthal, oleacein, and the phenolic alcohols. Beyond their role as reservoirs, they exhibit independent activity. Oleuropein attenuates DSS- and TNBS-induced colitis with dose-dependent reductions in disease activity index, colonic shortening, myeloperoxidase activity, COX-2 expression, and NF-κB activation [40,41]. Ex vivo treatment of inflamed colonic biopsies from ulcerative colitis patients with oleuropein decreases COX-2 protein, prostaglandin E2, and IL-17 [42], a rare bridge between animal mechanistic data and human IBD tissue. Oleuropein also remodels secondary bile acid profiles via gut microbiota and suppresses NLRP3 inflammasome activation, an axis directly relevant to IL-1β-mediated vascular inflammation [41].

### 6.3. Oleocanthal and Oleacein

These two dialdehydic secoiridoids deserve special emphasis in any IBD–cardiovascular argument because they touch the gut–endothelium–platelet axis at multiple nodes, and because their reactive aldehyde chemistry produces a pharmacological profile distinct from that of phenolic alcohols.

*Oleocanthal*, identified by Beauchamp et al. as the EVOO compound responsible for pharyngeal pungency, inhibits both COX-1 and COX-2 with potency approaching ibuprofen on a molar basis, doing so non-competitively rather than via the classical arachidonic-acid binding pocket—an unusual mechanism plausibly mediated by Schiff base formation with active-site lysine residues [29,30,67]. Its NSAID-like profile is functionally relevant in inflammatory contexts: in differentiated macrophages, oleocanthal suppresses LPS-induced NLRP3 inflammasome assembly, COX-2-dependent PGE2 production, TNF-α and IL-1β release, while activating the Nrf2/HO-1 antioxidant axis and inhibiting MAPKs (ERK, p38, JNK) [109]. Dietary oleocanthal supplementation reduces inflammation and oxidative stress in murine-collagen-induced arthritis [110] and modulates inflammation-related gene and miRNA-expression-including miR-155-5p, miR-34a-5p, and let-7c-5p-in human adipocytes by attenuating NF-κB activation, extending its mechanistic reach beyond direct enzyme inhibition to epigenomic regulation [32]. In silico screening across the OliveNet library confirms favourable COX docking and predicted membrane permeability without hERG channel liabilities [111]. The clinically translational claim, however, lies in platelets. Platelet COX-1 generates thromboxane A2, the principal autocrine amplifier of platelet activation; selective COX-1 inhibition is the mechanism by which low-dose aspirin confers cardiovascular benefit. In a randomised crossover trial in healthy men, acute ingestion of EVOO preparations differing in oleocanthal/oleacein composition reduced low-dose collagen-stimulated maximum platelet aggregation two hours after intake; the reduction in aggregation correlated best with oleocanthal intake, whereas inhibition of platelet-related eicosanoid production correlated more closely with total phenolic intake [43]. A subsequent postprandial study in type 2 diabetic patients reproduced reductions in P-selectin externalisation and aggregation [112]. A cross-sectional study in obese adults found higher self-reported olive oil intake inversely associated with multiple platelet activation markers in a dose–response pattern [113]. These data, although limited to acute outcomes and modest cohorts, establish the pharmacologically coherent bridge between oleocanthal’s biochemistry (COX-1/COX-2 inhibition) and a clinically meaningful surrogate (platelet activation) in populations with vascular risk profiles overlapping with IBD.

The ibuprofen-like profile of oleocanthal does, however, raise a legitimate safety question for IBD application, since classical NSAID exposure has historically been associated with disease flare. Two considerations temper this concern. First, current pharmacoepidemiological evidence does not support a clear causal NSAID–flare relationship in IBD: a large propensity-matched and self-controlled case series analysis of 35,031 IBD patients reported a prior event rate ratio of 0.95 (95% CI 0.89–1.01) for NSAID use and IBD exacerbation, suggesting that the apparent association is largely explained by reverse causality and residual confounding [114]. Second, the absolute quantity of oleocanthal delivered by realistic EVOO consumption (20–40 mL of an oleocanthal-rich oil at 100–800 mg/kg ≈ 2–32 mg/day) is well below the typical daily ibuprofen dose (1200–2400 mg), with non-linear and non-competitive COX kinetics that further differentiate it from classical NSAIDs. A clinical review specifically addressing olive oil components in IBD has nonetheless flagged the theoretical concern that very-high-phenolic oils could stimulate mucosal immunity and modestly raise CRP in some cohorts, and recommends that dose, oil characterisation, and disease activity be explicitly controlled in IBD interventional protocols [20].

*Oleacein*, oleocanthal’s catechol-bearing analogue, occupies an arguably more attractive pharmacological niche for an IBD–cardiovascular argument because it combines the reactive aldehyde chemistry of oleocanthal with the catechol antioxidant motif of hydroxytyrosol. It suppresses TLR4/MyD88/NF-κB signalling in LPS-challenged THP-1-derived macrophages, with downstream reductions in TNF-α, IL-6, IL-1β, and ROS generation [34]. The TLR4 axis is precisely the receptor system through which translocated LPS in IBD drives systemic endothelial activation, making this mechanism unusually relevant to the gut-to-vessel inflammation transfer described in Section 6. Oleacein attenuates inflammation in human adipocytes through NF-κB suppression and miRNA modulation, including downregulation of COX-2, VEGF, MMP-2, and MCP-1 [32]. Its glucuronide and sulfate metabolites retain COX-1 and COX-2 inhibition with low micromolar IC50 values, ensuring that the circulating species after ingestion remain pharmacologically credible rather than inert artefacts of conjugation [33]. Peripheral evidence from non-IBD models-including barrier preservation in experimental autoimmune encephalomyelitis and TrkB agonism with anti-neuroinflammatory effects—is consistent with the same mechanistic axes, but remains indirect and is therefore not weighted in the IBD–cardiovascular argument [115,116]. The combined profile relevant to this review—TLR4/NF-κB suppression, barrier protection in colitis-relevant epithelial systems, metabolite-retained COX inhibition, and direct antioxidant catechol activity—maps oleacein onto every major node of the gut–endothelium–platelet axis and arguably makes it the single most interesting compound in the EVOO matrix for the present clinical context.

### 6.4. Oleic Acid and Secondary Minor Components

Oleic acid contributes a metabolically favourable lipid background and modest endothelial benefits relative to saturated fat substitution, but it does not differentiate refined olive oil from high-phenolic EVOO. Tocopherols and squalene contribute to lipid phase oxidative protection; pentacyclic triterpenes (maslinic, oleanolic acid) add additional anti-inflammatory activity in preclinical settings, yet their concentrations and human pharmacokinetics do not warrant a central position in this argument. The phenolic fraction is what most plausibly differentiates the cardiovascular and intestinal bioactivity of high-phenolic EVOO from less bioactive olive oil products [10,11,16,35,36].

### 6.5. Shared and Distinct Mechanisms in Arterial Versus Venous Complications

A recurring conceptual hazard in the EVOO–cardiovascular literature is the implicit treatment of arterial atherothrombotic events (myocardial infarction, ischaemic stroke) and venous thromboembolic events (deep vein thrombosis, pulmonary embolism) as a single mechanistic package. They overlap, but they are not the same disease process, and the EVOO evidence base is not symmetric across them.

The arterial vascular phenotype in IBD is dominated by endothelial dysfunction, oxidised LDL accumulation, foam cell formation, plaque instability, and platelet COX-1-driven thromboxane generation at sites of plaque rupture [77,78,79,80,81,82,83,84,85,86,93,94,117]. Each of these nodes is mechanistically reachable by EVOO bioactives at exposures documented in non-IBD humans: high-phenolic EVOO reduces oxLDL and 8-isoprostane in randomised trials and meta-analyses, improves FMD and reduces soluble adhesion molecules, and oleocanthal-rich EVOO acutely attenuates platelet TXB2 and aggregation [8,16,35,36,37,43,112,113,118,119,120]. The arterial side of the EVOO argument is therefore comparatively well supported on surrogate markers, even in the absence of IBD-specific trials.

The venous thromboembolic phenotype, by contrast, is driven primarily by hypercoagulability, gut-derived LPS–TLR4 priming of tissue-factor-bearing microparticles, platelet–leukocyte aggregates, impaired fibrinolysis, and neutrophil extracellular traps providing a scaffold for erythrocyte and platelet adhesion in the contact-pathway-driven immunothrombotic cascade [95,96,97,98,99,102,103,104,105,117,121,122]. EVOO bioactives plausibly modulate upstream priming signals (TLR4/NF-κB suppression by oleacein, redox damping by hydroxytyrosol), but direct human evidence that EVOO modifies NETosis biomarkers, D-dimer dynamics, or VTE incidence is essentially absent. The mechanistic translation is therefore indirect on the venous side, and considerably more speculative than on the arterial side. Future trials in IBD should pre-specify which axis they intend to interrogate, with arterial surrogate panels (FMD, oxLDL, urinary 11-dehydro-TXB2) and venous surrogate panels (citrullinated histone H3, MPO–DNA complexes, soluble P-selectin, D-dimer) treated as separate domains.

## 7. Evidence Synthesis with a Strict Hierarchy

### 7.1. Human Studies in IBD with Cardiovascular Endpoints

None, to our knowledge, directly tests high-phenolic EVOO against vascular or platelet endpoints in IBD patients. In the Morvaridi crossover trial, EVOO was compared with canola oil in patients with ulcerative colitis. After 20 days of intervention, EVOO was associated with lower inflammatory markers and improved gastrointestinal symptoms, but endothelial, platelet, oxidative lipid, and thrombotic biomarkers were not assessed [123]. Ex vivo exposure of UC colonic biopsies to oleuropein produced anti-inflammatory effects on COX-2 and IL-17 [42], a tissue-level human signal but not a clinical outcome.

### 7.2. Human Studies Outside IBD with Vascular/Platelet/Oxidative Endpoints

This is where the strongest direct human evidence sits. The EUROLIVE crossover RCT (*n* = 200) demonstrated dose-dependent reductions in oxidised LDL and increases in HDL cholesterol with phenolic content of olive oil, providing the empirical foundation for the EU health claim [16,35]. Sub-studies showed concurrent rises in anti-oxLDL antibodies [36]. The SOLOS trial in stable coronary heart disease replicated the antioxidant signal [124]. The Valls trial showed that a phenolic-enriched virgin olive oil acutely improved postprandial endothelial function and reduced oxidised LDL in hypertensive patients, whereas Sánchez-Rodríguez et al. showed that virgin olive oils differing in phenolic and triterpene content modulated selected metabolic and endothelial risk biomarkers, particularly HDL cholesterol and endothelin-1, in healthy adults [118,125]. Schwingshackl’s meta-analysis of 32 RCTs found significant reductions in CRP, IL-6, E-selectin, and ICAM-1 with olive oil intake, with greater effects for EVOO than refined oil [119]. A dose–response meta-analysis reported greater reductions in oxidative stress biomarkers, particularly oxLDL and MDA, with higher olive oil phenolic content, although dose metrics should be interpreted cautiously because studies differ in oil dose, phenolic concentration, analytical method, and comparator oil [120]. PREDIMED demonstrated that a Mediterranean diet supplemented with EVOO at ~50 g/day reduced major cardiovascular events by ~30% over five years [126,127], with concurrent reductions in IL-6, ICAM-1, and MCP-1 over three years [128]; this trial, however, evaluated a dietary pattern rather than EVOO in isolation. On platelets, Agrawal et al. showed acute reduction in platelet aggregation after oleocanthal-rich EVOO in healthy men [43], and Katsa et al. extended this to type 2 diabetes [112]. Khandouzi et al. compared 25 mL/day polyphenol-rich EVOO with low-polyphenol refined olive oil for 6 weeks in patients undergoing coronary angiography and reported reductions in LDL-C and CRP, together with increased ex vivo LPS-stimulated IL-10 production [129]. Hernáez et al. demonstrated reduced LDL atherogenicity (electronegativity) with high-phenolic EVOO [37].

### 7.3. Animal and Ex Vivo IBD/Colitis Models

Robust, multi-model preclinical evidence supports anti-colitic activity of oleuropein, hydroxytyrosol, oleocanthal-rich EVOO, and oleacein, with consistent mechanisms (NF-κB, COX-2, NLRP3, tight junction restoration, microbiome modulation) [39,40,41,116,130,131].

### 7.4. In Vitro Mechanistic Studies

Extensive coherent data on COX inhibition, NF-κB and TLR4 suppression, Nrf2/HO-1 activation, NLRP3 modulation, and barrier protection across endothelial, epithelial, macrophage, and platelet systems were available [22,23,24,32,33,34,38,107,108,109,111,115].

The cumulative inference is that the molecular plausibility of EVOO secoiridoids and phenolic alcohols acting on the gut–endothelium–platelet axis is strong; non-IBD human evidence for vascular, lipid oxidation, inflammatory, and platelet outcomes with high-phenolic EVOO is moderate to strong; and IBD-specific human evidence with cardiovascular endpoints is absent or remains preliminary. This stratified verdict must constrain any clinical recommendation.

A further methodological consideration concerns the phenolic content of the comparator and intervention oils. Several trials nominally testing “olive oil” used products whose hydroxytyrosol-equivalent content fell below the EUROLIVE high-phenolic threshold (~366 mg/kg total phenolics, of which a defined fraction is hydroxytyrosol-derivatives), making null findings difficult to interpret as evidence of inactivity rather than as evidence of insufficient dose [17,18,120,124]. The corollary is that null trials of “olive oil” cannot be cited against high-phenolic EVOO without re-examining the chemical characterisation of the test oil. Similarly, several positive vascular and platelet trials used oleocanthal- or hydroxytyrosol-enriched oils that, in commercial terms, would be classed as boutique high-phenolic monovarietals; their effects therefore inform what can be achieved with chemically defined high-phenolic EVOO, not what an average supermarket bottle delivers [28,31,37,43,112]. Replication and dose-finding in larger, real-world EVOO populations are an explicit research priority.

## 8. Human Outcome Domains: What Translates and What Does Not

For the most relevant cardiovascular surrogate outcomes, the human evidence base for high-phenolic EVOO can be summarised with reasonable precision.

Endothelial function (FMD, biomarkers): Consistent improvement with high-phenolic vs. low-phenolic oils in hypertensive and healthy populations; no IBD-specific trial [118,125].

Arterial stiffness (cfPWV/AIx): IBD cohorts show higher cfPWV and AIx, supporting aortic stiffening as an inflammation-linked vascular phenotype. However, translation to EVOO is unproven: the OLIVAUS high-phenolic EVOO crossover trial found no significant PWV/AIx change after 3 weeks. Thus, PWV should remain an exploratory, longer term IBD-specific endpoint [80,82,93].

Oxidised LDL and lipid peroxidation: The strongest signal-dose-dependent reductions across multiple RCTs and meta-analyses, anchoring the EU health claim [16,35,36,37,120]; not directly tested in IBD.

Inflammatory biomarkers (CRP, IL-6, TNF-α): Consistent reductions with EVOO interventions in mixed populations, including the only modest IBD RCT [119,123,125,129].

Platelet activation (TXB2, P-selectin, aggregation): Direct evidence of acute attenuation by oleocanthal-rich EVOO in healthy and diabetic populations [43,112,113]; not tested in IBD; longer term sustained effects on platelet phenotype, NETosis markers, and platelet–leukocyte aggregates not established. Because IBD platelets are constitutively hyperreactive [95,96,97,98,99], the absolute magnitude of any oleocanthal-driven attenuation could plausibly differ from that observed in healthy volunteers, in either direction—a question only an IBD-specific trial can resolve.

Hard cardiovascular events: Only PREDIMED provides RCT-level data, and only at the dietary pattern level; no IBD-specific event trial exists [126,127].

Intestinal outcomes specifically: In non-IBD populations, EVOO and isolated phenolics have shown mucosal anti-inflammatory effects in functional bowel disorders and irritable bowel models, but in IBD the only randomised intervention with EVOO is the Morvaridi trial in active ulcerative colitis was associated with reductions in hs-CRP and GSRS symptom scores relative to canola oil control [123]. The trial was modest in size and did not pre-register vascular biomarkers; nonetheless it demonstrates feasibility of EVOO administration at a relevant dose in active IBD, an important practical point for the next generation of cardiovascular endpoint trials.

Taken together, the evidence is strongest for surrogate markers and weakest where the clinical question is most specific to IBD. The direction of effect across surrogate domains, however, is consistent: high-phenolic EVOO interventions move oxidative, inflammatory, vascular, and platelet biomarkers in the cardioprotective direction in non-IBD populations, providing a coherent biological prior against which IBD-specific trials can be powered. The required leap is therefore one of population, not of mechanism. Given the heterogeneity of available evidence, Table 2 summarises which EVOO-related cardiovascular surrogate outcomes are currently supported by human data, and which remain only indirectly translatable to IBD-associated cardiovascular risk.

## 9. Conclusions

High-phenolic extra-virgin olive oil is not interchangeable with “olive oil.” It is a chemically distinct bioactive matrix in which a small phenolic fraction—dominated by oleuropein- and ligstroside-derived secoiridoids, the dialdehydic congeners oleocanthal and oleacein, and the phenolic alcohols hydroxytyrosol and tyrosol—carries a pharmacological signal that plausibly intersects the gut–endothelium–platelet axis implicated in cardiovascular complications of inflammatory bowel disease. Mechanistic evidence from in vitro and animal systems supports plausible activity at every node of this axis: barrier protection by hydroxytyrosol and oleacein; TLR4/NF-κB suppression by oleacein; cyclooxygenase inhibition by oleocanthal; Nrf2/HO-1 activation by hydroxytyrosol; tight junction restoration in colitis models by oleuropein and hydroxytyrosol; and acute platelet attenuation in human postprandial trials of oleocanthal-rich EVOO. Non-IBD human trials add credible support for endothelial, oxidative LDL, and inflammatory benefit, anchored by the EUROLIVE dose–response and the EU health claim for hydroxytyrosol and its derivatives.

Yet the asymmetry is decisive: direct interventional evidence in IBD patients with cardiovascular surrogate or hard endpoints is essentially absent. Existing IBD trials have measured intestinal outcomes; existing cardiovascular trials have studied non-IBD populations. The molecular plausibility is strong; the IBD-specific clinical proof is not. The next decade should be defined by chemically characterised, biomarker-anchored trials in IBD populations of the kind outlined above. Until those are completed, high-phenolic EVOO should be discussed in IBD–cardiovascular contexts as a biologically coherent candidate intervention, not a validated therapy.

## Figures and Tables

**Table 1:** Major EVOO phenolics relevant to IBD-associated vascular risk.

Compound Group	Chemical Origin/Main Metabolites	Main Biological Targets	Key References
Hydroxytyrosol; tyrosol	Phenolic alcohols derived from secoiridoids; mainly sulfate, glucuronide, methylated, and microbial metabolites	Nrf2/ARE, NF-κB, epithelial/endothelial barrier, oxLDL protection	[16,22,38,39]
Oleuropein- and ligstroside-derived aglycones	Secoiridoid aglycones from olive fruit glycosides; hydrolysed to hydroxytyrosol/tyrosol	COX-2, NF-κB, NLRP3, microbiota/bile acid modulation	[25,28,40,41,42]
Oleocanthal; oleacein	Dialdehydic secoiridoids; form amine/glycine adducts and phase II metabolites	COX-1/2, TLR4/MyD88/NF-κB, platelet activation, ROS, barrier-related inflammation	[29,33,34,43]

Abbreviations: ARE, antioxidant response element; COX, cyclooxygenase; EVOO, extra-virgin olive oil; IBD, inflammatory bowel disease; MyD88, myeloid differentiation primary response 88; NF-κB, nuclear factor kappa-light-chain-enhancer of activated B cells; NLRP3, NOD-like receptor family pyrin domain-containing 3; Nrf2, nuclear factor erythroid 2-related factor 2; oxLDL, oxidised low-density lipoprotein; ROS, reactive oxygen species; TLR4, toll-like receptor 4.

**Table 2:** Translational evidence linking high-phenolic EVOO to cardiovascular risk domains relevant to inflammatory bowel disease.

Outcome Domain	Human EVOO Evidence	IBD/CVD Translation	Key References
Endothelial function	High-phenolic or phenol-enriched olive oil improves endothelial function in non-IBD human studies.	Relevant surrogate, but no IBD-specific EVOO vascular trial.	[118,119,125]
Arterial stiffness	IBD is associated with higher cfPWV/AIx, but high-polyphenol EVOO did not improve arterial stiffness over 3 weeks in healthy adults.	Use cfPWV/AIx only as exploratory, longer term IBD endpoint.	[80,82,93,132]
Oxidative stress/oxLDL	Strongest translational signal: olive oil phenols reduce oxLDL, MDA, and LDL atherogenicity.	Mechanistically relevant to IBD-CVD risk but not directly tested in IBD.	[35,36,37,120]
Inflammatory biomarkers	EVOO/olive oil interventions show modest anti-inflammatory effects; the UC EVOO trial mainly supports feasibility and preliminary inflammation/GI symptom benefit.	Do not overclaim cytokine effects until replicated in IBD trials.	[119,123,125,128,129]
Platelet activation	Oleocanthal-rich EVOO acutely reduces platelet aggregation in healthy and T2DM populations.	Promising, but no IBD platelet trial; avoid claiming sustained antithrombotic benefit.	[43,95,96,97,98,99,112,113]
Hard CVD events	PREDIMED supports event reduction at Mediterranean diet pattern level, not as isolated EVOO pharmacology.	No IBD-specific cardiovascular endpoint trial exists.	[126,127]

Abbreviations: AIx, augmentation index; cfPWV, carotid–femoral pulse wave velocity; CVD, cardiovascular disease; EVOO, extra-virgin olive oil; GI, gastrointestinal; IBD, inflammatory bowel disease; LDL, low-density lipoprotein; MDA, malondialdehyde; oxLDL, oxidised low-density lipoprotein; PREDIMED, Prevención con Dieta Mediterránea; PWV, pulse wave velocity; T2DM, type 2 diabetes mellitus; UC, ulcerative colitis.

## Data Availability

No new data were created or analyzed in this study.

## Abbreviations

The following abbreviations are used in this manuscript:

11-dehydro-TXB2	11-dehydro-thromboxane B2
AIx	augmentation index
ARE	antioxidant response element
ATE	arterial thromboembolism
CD40L	CD40 ligand
cfPWV	carotid–femoral pulse wave velocity
CI	confidence interval
citH3	citrullinated histone H3
COX	cyclooxygenase
COX-1	cyclooxygenase-1
COX-2	cyclooxygenase-2
CRP	C-reactive protein
CVD	cardiovascular disease
CYP72	cytochrome P450 family 72
DSS	dextran sulfate sodium
EAE	experimental autoimmune encephalomyelitis
EFSA	European Food Safety Authority
eNOS	endothelial nitric oxide synthase
ERK	extracellular signal-regulated kinase
EU	European Union
EUROLIVE	Effect of Olive Oil Consumption on Oxidative Damage in European Populations
EVOO	extra-virgin olive oil
FMD	flow-mediated dilation
GCLC	glutamate-cysteine ligase catalytic subunit
GI	gastrointestinal
HDL	high-density lipoprotein
hERG	human ether-à-go-go-related gene
HO-1	heme oxygenase-1
hs-CRP	high-sensitivity C-reactive protein
HUVEC	human umbilical vein endothelial cell
IBD	inflammatory bowel disease
IC50	half-maximal inhibitory concentration
ICAM-1	intercellular adhesion molecule-1
IL	interleukin
IL-1β	interleukin-1 beta
IL-6	interleukin-6
IL-8	interleukin-8
IL-17	interleukin-17
IOC	International Olive Council
JAK	Janus kinase
JNK	c-Jun N-terminal kinase
LDL	low-density lipoprotein
LPS	lipopolysaccharide
MAPK	mitogen-activated protein kinase
MCP-1	monocyte chemoattractant protein-1
MDA	malondialdehyde
MEDLINE	Medical Literature Analysis and Retrieval System Online
miRNA	microRNA
MMP-2	matrix metalloproteinase-2
MPO–DNA	myeloperoxidase–DNA complex
MyD88	myeloid differentiation primary response 88
NADPH	nicotinamide adenine dinucleotide phosphate
NET/NETs	neutrophil extracellular trap(s)
NF-κB	nuclear factor kappa-light-chain-enhancer of activated B cells
NLRP3	NOD-like receptor family pyrin domain-containing 3
NO	nitric oxide
NQO1	NAD(P)H quinone oxidoreductase 1
Nrf2	nuclear factor erythroid 2-related factor 2
NSAID	non-steroidal anti-inflammatory drug
OeEAME1/2	*Olea europaea* elenolic acid methylesterase 1 and 2
OeGLU	*Olea europaea* oleuropein β-glucosidase
OeISY	*Olea europaea* iridoid synthase
OeOMES	*Olea europaea* oleoside methyl ester synthase
OeSXS	*Olea europaea* secoxyloganin synthase
OLIVAUS	high-polyphenol extra-virgin olive oil trial in healthy Australian adults
oxLDL	oxidised low-density lipoprotein
PGE2	prostaglandin E2
PREDIMED	Prevención con Dieta Mediterránea
PWV	pulse wave velocity
RANTES	regulated upon activation, normal T-cell expressed and secreted
RCT	randomised controlled trial
ROS	reactive oxygen species
SANRA	Scale for the Assessment of Narrative Review Articles
SCFA	short-chain fatty acid
SCCAI	Simple Clinical Colitis Activity Index
sICAM-1	soluble intercellular adhesion molecule-1
SOLOS	Spanish Olive Oil Study
sVCAM-1	soluble vascular cell adhesion molecule-1
THP-1	human monocytic leukaemia cell line
TLR4	Toll-like receptor 4
TNBS	2,4,6-trinitrobenzene sulfonic acid
TNF-α	tumour necrosis factor-alpha
TXA2	thromboxane A2
TXB2	thromboxane B2
UC	ulcerative colitis
VCAM-1	vascular cell adhesion molecule-1
VEGF	vascular endothelial growth factor
VTE	venous thromboembolism
ZO-1	zonula occludens-1

## References

[1] Thomas D.-R., Huangfu G., Yeaman F., Sukudom S., Lan N.S.R., Dwivedi G., Thin L. (2025). Association between Inflammatory Bowel Disease, Current Therapies, and Cardiovascular Events: A Review and Meta-Analysis of Data from 2.2 Million Individuals. J. Crohn’s Colitis.

[2] Zaka A., Mridha N., Subhaharan D., Jones M., Niranjan S., Mohsen W., Ramaswamy P.K. (2023). Inflammatory Bowel Disease Patients Have an Increased Risk of Acute Coronary Syndrome: A Systematic Review and Meta-Analysis. Open Heart.

[3] Feng W., Chen G., Cai D., Zhao S., Cheng J., Shen H. (2017). Inflammatory Bowel Disease and Risk of Ischemic Heart Disease: An Updated Meta-Analysis of Cohort Studies. J. Am. Heart Assoc..

[4] Luo C., Liu L., Zhu D., Ge Z., Chen Y., Chen F. (2025). Risk of Stroke in Patients with Inflammatory Bowel Disease: A Systematic Review and Meta-Analysis. BMC Gastroenterol..

[5] Goyal A., Tariq M.D., Hurjkaliani S., Maryyum A., Thakkar K., Thakur T., Dahiya D.S. (2025). Is the Risk of Heart Failure Increased in Patients with Inflammatory Bowel Disease? A Meta-Analysis. J. Investig. Med..

[6] Sleutjes J.A.M., Van Lennep J.E.R., Van Der Woude C.J., De Vries A.C. (2021). Thromboembolic and Atherosclerotic Cardiovascular Events in Inflammatory Bowel Disease: Epidemiology, Pathogenesis and Clinical Management. Ther. Adv. Gastroenterol..

[7] Papa A., Santini P., De Lucia S.S., Maresca R., Porfidia A., Pignatelli P., Gasbarrini A., Violi F., Pola R. (2023). Gut Dysbiosis-Related Thrombosis in Inflammatory Bowel Disease: Potential Disease Mechanisms and Emerging Therapeutic Strategies. Thromb. Res..

[8] Sanchez Cruz C., Rojas Huerta A., Lima Barrientos J., Rodriguez C., Devani A., Boosahda V., Rasagna Mareddy N.S., Briceno Silva G., Del Castillo Miranda J.C., Reyes Gochi K.A. (2024). Inflammatory Bowel Disease and Cardiovascular Disease: An Integrative Review with a Focus on the Gut Microbiome. Cureus.

[9] Šantić R., Pavlović N., Kumrić M., Vilović M., Božić J. (2025). Pathophysiological Links Between Inflammatory Bowel Disease and Cardiovascular Disease: The Role of Dysbiosis and Emerging Biomarkers. Biomedicines.

[10] Jimenez-Lopez C., Carpena M., Lourenço-Lopes C., Gallardo-Gomez M., Lorenzo J.M., Barba F.J., Prieto M.A., Simal-Gandara J. (2020). Bioactive Compounds and Quality of Extra Virgin Olive Oil. Foods.

[11] Bendini A., Cerretani L., Carrasco-Pancorbo A., Gómez-Caravaca A.M., Segura-Carretero A., Fernández-Gutiérrez A., Lercker G. (2007). Phenolic Molecules in Virgin Olive Oils: A Survey of Their Sensory Properties, Health Effects, Antioxidant Activity and Analytical Methods. An Overview of the Last Decade Alessandra. Molecules.

[12] Servili M., Esposto S., Fabiani R., Urbani S., Taticchi A., Mariucci F., Selvaggini R., Montedoro G.F. (2009). Phenolic Compounds in Olive Oil: Antioxidant, Health and Organoleptic Activities According to Their Chemical Structure. Inflammopharmacology.

[13] Tripoli E., Giammanco M., Tabacchi G., Di Majo D., Giammanco S., La Guardia M. (2005). The Phenolic Compounds of Olive Oil: Structure, Biological Activity and Beneficial Effects on Human Health. Nutr. Res. Rev..

[14] Cicerale S., Lucas L., Keast R. (2010). Biological Activities of Phenolic Compounds Present in Virgin Olive Oil. Int. J. Mol. Sci..

[15] Gorzynik-Debicka M., Przychodzen P., Cappello F., Kuban-Jankowska A., Marino Gammazza A., Knap N., Wozniak M., Gorska-Ponikowska M. (2018). Potential Health Benefits of Olive Oil and Plant Polyphenols. Int. J. Mol. Sci..

[16] EFSA Panel on Dietetic Products, Nutrition and Allergies (NDA) (2011). Scientific Opinion on the Substantiation of Health Claims Related to Polyphenols in Olive and Protection of LDL Particles from Oxidative Damage (ID 1333, 1638, 1639, 1696, 2865), Maintenance of Normal Blood HDL Cholesterol Concentrations (ID 1639), Maintenance of Normal Blood Pressure (ID 3781), “Anti-inflammatory Properties” (ID 1882), “Contributes to the Upper Respiratory Tract Health” (ID 3468), “Can Help to Maintain a Normal Function of Gastrointestinal Tract” (3779), and “Contributes to Body Defences against External Agents” (ID 3467) Pursuant to Article 13(1) of Regulation (EC) No 1924/2006. EFSA.

[17] Mastralexi A., Nenadis N., Tsimidou M.Z. (2014). Addressing Analytical Requirements To Support Health Claims on “Olive Oil Polyphenols” (EC Regulation 432/2012). J. Agric. Food Chem..

[18] Bellumori M., Cecchi L., Innocenti M., Clodoveo M.L., Corbo F., Mulinacci N. (2019). The EFSA Health Claim on Olive Oil Polyphenols: Acid Hydrolysis Validation and Total Hydroxytyrosol and Tyrosol Determination in Italian Virgin Olive Oils. Molecules.

[19] Millman J.F., Okamoto S., Teruya T., Uema T., Ikematsu S., Shimabukuro M., Masuzaki H. (2021). Extra-Virgin Olive Oil and the Gut-Brain Axis: Influence on Gut Microbiota, Mucosal Immunity, and Cardiometabolic and Cognitive Health. Nutr. Rev..

[20] Vrdoljak J., Kumric M., Vilovic M., Martinovic D., Tomic I.J., Krnic M., Ticinovic Kurir T., Bozic J. (2022). Effects of Olive Oil and Its Components on Intestinal Inflammation and Inflammatory Bowel Disease. Nutrients.

[21] Krichene D., Salvador M.D., Fregapane G. (2015). Stability of Virgin Olive Oil Phenolic Compounds during Long-Term Storage (18 Months) at Temperatures of 5–50 °C. J. Agric. Food Chem..

[22] Serreli G., Deiana M. (2018). Biological Relevance of Extra Virgin Olive Oil Polyphenols Metabolites. Antioxidants.

[23] Velotti F., Bernini R. (2023). Hydroxytyrosol Interference with Inflammaging via Modulation of Inflammation and Autophagy. Nutrients.

[24] Batarfi W.A., Yunus M.H.M., Hamid A.A., Lee Y.T., Maarof M. (2024). Hydroxytyrosol: A Promising Therapeutic Agent for Mitigating Inflammation and Apoptosis. Pharmaceutics.

[25] Obied H.K., Prenzler P.D., Ryan D., Servili M., Taticchi A., Esposto S., Robards K. (2008). Biosynthesis and Biotransformations of Phenol-Conjugated Oleosidic Secoiridoids from *Olea europaea* L.. Nat. Prod. Rep..

[26] Montedoro G., Servili M., Baldioli M., Selvaggini R., Miniati E., Macchioni A. (1993). Simple and Hydrolyzable Compounds in Virgin Olive Oil. 3. Spectroscopic Characterizations of the Secoiridoid Derivatives. J. Agric. Food Chem..

[27] Rodríguez-López C.E., Hong B., Paetz C., Nakamura Y., Koudounas K., Passeri V., Baldoni L., Alagna F., Calderini O., O’Connor S.E. (2021). Two Bi-functional Cytochrome P450 CYP72 Enzymes from Olive (*Olea Europaea*) Catalyze the Oxidative C-C Bond Cleavage in the Biosynthesis of Secoxy-iridoids—Flavor and Quality Determinants in Olive Oil. New Phytol..

[28] Olmo-Cunillera A., Pérez M., López-Yerena A., Abuhabib M.M., Ninot A., Romero-Aroca A., Vallverdú-Queralt A., Lamuela-Raventós R.M. (2023). Oleacein and Oleocanthal: Key Metabolites in the Stability of Extra Virgin Olive Oil. Antioxidants.

[29] Beauchamp G.K., Keast R.S.J., Morel D., Lin J., Pika J., Han Q., Lee C.-H., Smith A.B., Breslin P.A.S. (2005). Ibuprofen-like Activity in Extra-Virgin Olive Oil. Nature.

[30] Smith A.B., Han Q., Breslin P.A.S., Beauchamp G.K. (2005). Synthesis and Assignment of Absolute Configuration of (−)-Oleocanthal: A Potent, Naturally Occurring Non-Steroidal Anti-Inflammatory and Anti-Oxidant Agent Derived from Extra Virgin Olive Oils. Org. Lett..

[31] López-Yerena A., Ninot A., Jiménez-Ruiz N., Lozano-Castellón J., Pérez M., Escribano-Ferrer E., Romero-Aroca A., Lamuela-Raventós R.M., Vallverdú-Queralt A. (2021). Influence of the Ripening Stage and Extraction Conditions on the Phenolic Fingerprint of ‘Corbella’ Extra-Virgin Olive Oil. Antioxidants.

[32] Carpi S., Scoditti E., Massaro M., Polini B., Manera C., Digiacomo M., Esposito Salsano J., Poli G., Tuccinardi T., Doccini S. (2019). The Extra-Virgin Olive Oil Polyphenols Oleocanthal and Oleacein Counteract Inflammation-Related Gene and miRNA Expression in Adipocytes by Attenuating NF-κB Activation. Nutrients.

[33] Costa V., Costa M., Videira R.A., Andrade P.B., Paiva-Martins F. (2022). Anti-Inflammatory Activity of Olive Oil Polyphenols—The Role of Oleacein and Its Metabolites. Biomedicines.

[34] Cirmi S., Maugeri A., Russo C., Musumeci L., Navarra M., Lombardo G.E. (2022). Oleacein Attenuates Lipopolysaccharide-Induced Inflammation in THP-1-Derived Macrophages by the Inhibition of TLR4/MyD88/NF-κB Pathway. Int. J. Mol. Sci..

[35] Covas M.-I., Nyyssönen K., Poulsen H.E., Kaikkonen J., Zunft H.-J.F., Kiesewetter H., Gaddi A., De La Torre R., Mursu J., Bäumler H. (2006). The Effect of Polyphenols in Olive Oil on Heart Disease Risk Factors: A Randomized Trial. Ann. Intern. Med..

[36] Castañer O., Fitó M., López-Sabater M.C., Poulsen H.E., Nyyssönen K., Schröder H., Salonen J.T., De La Torre-Carbot K., Zunft H.-F., De La Torre R. (2011). The Effect of Olive Oil Polyphenols on Antibodies against Oxidized LDL. A Randomized Clinical Trial. Clin. Nutr..

[37] Hernáez Á., Remaley A.T., Farràs M., Fernández-Castillejo S., Subirana I., Schröder H., Fernández-Mampel M., Muñoz-Aguayo D., Sampson M., Solà R. (2015). Olive Oil Polyphenols Decrease LDL Concentrations and LDL Atherogenicity in Men in a Randomized Controlled Trial. J. Nutr..

[38] Zodio S., Serreli G., Melis M.P., Franchi B., Boronat A., De La Torre R., Deiana M. (2024). Protective Effect of Hydroxytyrosol and Tyrosol Metabolites in LPS-Induced Vascular Barrier Derangement In Vitro. Front. Nutr..

[39] Wang Q., Wang C., Abdullah, Tian W., Qiu Z., Song M., Cao Y., Xiao J. (2022). Hydroxytyrosol Alleviates Dextran Sulfate Sodium-Induced Colitis by Modulating Inflammatory Responses, Intestinal Barrier, and Microbiome. J. Agric. Food Chem..

[40] Giner E., Andújar I., Recio M.C., Ríos J.L., Cerdá-Nicolás J.M., Giner R.M. (2011). Oleuropein Ameliorates Acute Colitis in Mice. J. Agric. Food Chem..

[41] Zang R., Zhou R., Li Y., Liu Z., Wu H., Lu L., Xu H. (2025). Oleuropein Regulates Bile Acid Metabolism via Modulating the Gut Microbiota, Thereby Alleviating DSS-Induced Ulcerative Colitis in Mice. Foods.

[42] Larussa T., Oliverio M., Suraci E., Greco M., Placida R., Gervasi S., Marasco R., Imeneo M., Paolino D., Tucci L. (2017). Oleuropein Decreases Cyclooxygenase-2 and Interleukin-17 Expression and Attenuates Inflammatory Damage in Colonic Samples from Ulcerative Colitis Patients. Nutrients.

[43] Agrawal K., Melliou E., Li X., Pedersen T.L., Wang S.C., Magiatis P., Newman J.W., Holt R.R. (2017). Oleocanthal-Rich Extra Virgin Olive Oil Demonstrates Acute Anti-Platelet Effects in Healthy Men in a Randomized Trial. J. Funct. Foods.

[44] Alagna F., Mariotti R., Panara F., Caporali S., Urbani S., Veneziani G., Esposto S., Taticchi A., Rosati A., Rao R. (2012). Olive Phenolic Compounds: Metabolic and Transcriptional Profiling during Fruit Development. BMC Plant Biol..

[45] Koudounas K., Thomopoulou M., Rigakou A., Angeli E., Melliou E., Magiatis P., Hatzopoulos P. (2021). Silencing of Oleuropein β-Glucosidase Abolishes the Biosynthetic Capacity of Secoiridoids in Olives. Front. Plant Sci..

[46] Servili M., Montedoro G. (2002). Contribution of Phenolic Compounds to Virgin Olive Oil Quality. Eur. J. Lipid Sci. Technol..

[47] Ramírez E., Medina E., Brenes M., Romero C. (2014). Endogenous Enzymes Involved in the Transformation of Oleuropein in Spanish Table Olive Varieties. J. Agric. Food Chem..

[48] Volk J., Sarafeddinov A., Unver T., Marx S., Tretzel J., Zotzel J., Warzecha H. (2019). Two Novel Methylesterases from Olea Europaea Contribute to the Catabolism of Oleoside-Type Secoiridoid Esters. Planta.

[49] Mousavi S., Mariotti R., Stanzione V., Pandolfi S., Mastio V., Baldoni L., Cultrera N.G.M. (2021). Evolution of Extra Virgin Olive Oil Quality under Different Storage Conditions. Foods.

[50] Cicerale S., Conlan X.A., Barnett N.W., Sinclair A.J., Keast R.S.J. (2009). Influence of Heat on Biological Activity and Concentration of Oleocanthal—A Natural Anti-Inflammatory Agent in Virgin Olive Oil. J. Agric. Food Chem..

[51] Miró-Casas E., Farré Albaladejo M., Covas M.-I., Rodriguez J.O., Menoyo Colomer E., Lamuela Raventós R.M., De La Torre R. (2001). Capillary Gas Chromatography–Mass Spectrometry Quantitative Determination of Hydroxytyrosol and Tyrosol in Human Urine after Olive Oil Intake. Anal. Biochem..

[52] Miro-Casas E., Covas M.-I., Farre M., Fito M., Ortuño J., Weinbrenner T., Roset P., De La Torre R. (2003). Hydroxytyrosol Disposition in Humans. Clin. Chem..

[53] Miró-Casas E., Covas M.-I., Fitó M., Farré-Albadalejo M., Marrugat J., De La Torre R. (2003). Tyrosol and Hydroxytyrosol Are Absorbed from Moderate and Sustained Doses of Virgin Olive Oil in Humans. Eur. J. Clin. Nutr..

[54] Miró Casas E., Farré Albadalejo M., Covas Planells M.I., Fitó Colomer M., Lamuela Raventós R.M., De La Torre Fornell R. (2001). Tyrosol Bioavailability in Humans after Ingestion of Virgin Olive Oil. Clin. Chem..

[55] Visioli F., Galli C., Grande S., Colonnelli K., Patelli C., Galli G., Caruso D. (2003). Hydroxytyrosol Excretion Differs between Rats and Humans and Depends on the Vehicle of Administration. J. Nutr..

[56] Alemán-Jiménez C., Domínguez-Perles R., Medina S., Prgomet I., López-González I., Simonelli-Muñoz A., Campillo-Cano M., Auñón D., Ferreres F., Gil-Izquierdo Á. (2021). Pharmacokinetics and Bioavailability of Hydroxytyrosol Are Dependent on the Food Matrix in Humans. Eur. J. Nutr..

[57] Bender C., Strassmann S., Golz C. (2023). Oral Bioavailability and Metabolism of Hydroxytyrosol from Food Supplements. Nutrients.

[58] Galmés S., Reynés B., Palou M., Palou-March A., Palou A. (2021). Absorption, Distribution, Metabolism, and Excretion of the Main Olive Tree Phenols and Polyphenols: A Literature Review. J. Agric. Food Chem..

[59] Nikou T., Sakavitsi M.E., Kalampokis E., Halabalaki M. (2022). Metabolism and Bioavailability of Olive Bioactive Constituents Based on In Vitro, In Vivo and Human Studies. Nutrients.

[60] Kundisová I., Colom H., Juan M.E., Planas J.M. (2024). Pharmacokinetics of Hydroxytyrosol and Its Sulfate and Glucuronide Metabolites after the Oral Administration of Table Olives to Sprague-Dawley Rats. J. Agric. Food Chem..

[61] Caruso D., Visioli F., Patelli R., Galli C., Galli G. (2001). Urinary Excretion of Olive Oil Phenols and Their Metabolites in Humans. Metabolism.

[62] Khymenets O., Fitó M., Touriño S., Muñoz-Aguayo D., Pujadas M., Torres J.L., Joglar J., Farré M., Covas M.-I., De La Torre R. (2010). Antioxidant Activities of Hydroxytyrosol Main Metabolites Do Not Contribute to Beneficial Health Effects after Olive Oil Ingestion. Drug Metab. Dispos..

[63] Mosele J.I., Martín-Peláez S., Macià A., Farràs M., Valls R., Catalán Ú., Motilva M. (2014). Faecal Microbial Metabolism of Olive Oil Phenolic Compounds: In Vitro and In Vivo Approaches. Mol. Nutr. Food Res..

[64] Rocchetti G., Luisa Callegari M., Senizza A., Giuberti G., Ruzzolini J., Romani A., Urciuoli S., Nediani C., Lucini L. (2022). Oleuropein from Olive Leaf Extracts and Extra-Virgin Olive Oil Provides Distinctive Phenolic Profiles and Modulation of Microbiota in the Large Intestine. Food Chem..

[65] Andújar-Tenorio N., Cobo A., Martínez-Rodríguez A.M., Hidalgo M., Prieto I., Gálvez A., Martínez-Cañamero M. (2023). Intestinal Microbiota Modulation at the Strain Level by the Olive Oil Polyphenols in the Diet. Front. Nutr..

[66] Sakavitsi M.E., Breynaert A., Nikou T., Lauwers S., Pieters L., Hermans N., Halabalaki M. (2022). Availability and Metabolic Fate of Olive Phenolic Alcohols Hydroxytyrosol and Tyrosol in the Human GI Tract Simulated by the In Vitro GIDM–Colon Model. Metabolites.

[67] Darakjian L.I., Rigakou A., Brannen A., Qusa M.H., Tasiakou N., Diamantakos P., Reed M.N., Panizzi P., Boersma M.D., Melliou E. (2021). Spontaneous In Vitro and In Vivo Interaction of (−)-Oleocanthal with Glycine in Biological Fluids: Novel Pharmacokinetic Markers. ACS Pharmacol. Transl. Sci..

[68] Di Risola D., Laurenti D., Ferraro F., Ciogli A., Manetto S., Gazzilli Y., Federico R., Francioso A., Mosca L., Mattioli R. (2025). Spontaneous Reaction of Oleacein and Oleocanthal with Primary Amines: A Biochemical Perspective. Molecules.

[69] López-Yerena A., Vallverdú-Queralt A., Jáuregui O., Garcia-Sala X., Lamuela-Raventós R.M., Escribano-Ferrer E. (2021). Tissue Distribution of Oleocanthal and Its Metabolites after Oral Ingestion in Rats. Antioxidants.

[70] López-Yerena A., Vallverdú-Queralt A., Mols R., Augustijns P., Lamuela-Raventós R.M., Escribano-Ferrer E. (2020). Absorption and Intestinal Metabolic Profile of Oleocanthal in Rats. Pharmaceutics.

[71] Lozano-Castellón J., López-Yerena A., Rinaldi De Alvarenga J.F., Romero Del Castillo-Alba J., Vallverdú-Queralt A., Escribano-Ferrer E., Lamuela-Raventós R.M. (2020). Health-Promoting Properties of Oleocanthal and Oleacein: Two Secoiridoids from Extra-Virgin Olive Oil. Crit. Rev. Food Sci. Nutr..

[72] Rodríguez-Morató J., Xicota L., Fitó M., Farré M., Dierssen M., De La Torre R. (2015). Potential Role of Olive Oil Phenolic Compounds in the Prevention of Neurodegenerative Diseases. Molecules.

[73] Jordán M., García-Acosta N., Espartero J.L., Goya L., Mateos R. (2025). Hydroxytyrosol Bioavailability: Unraveling Influencing Factors and Optimization Strategies for Dietary Supplements. Nutrients.

[74] Montoro-Alonso S., Duque-Soto C., Rueda-Robles A., Reina-Manuel J., Quirantes-Piné R., Borrás-Linares I., Lozano-Sánchez J. (2024). Functional Olive Oil Production via Emulsions: Evaluation of Phenolic Encapsulation Efficiency, Storage Stability, and Bioavailability. Nutrients.

[75] Meyer A., Agrawal M., Savin-Shalom E., Wong E.C.L., Levinson C., Gold S., Narula N., Colombel J.-F., Carbonnel F. (2025). Impact of Diet on Inflammatory Bowel Disease Risk: Systematic Review, Meta-Analyses and Implications for Prevention. eClinicalMedicine.

[76] El Amrousy D., Elashry H., Salamah A., Maher S., Abd-Elsalam S.M., Hasan S. (2022). Adherence to the Mediterranean Diet Improved Clinical Scores and Inflammatory Markers in Children with Active Inflammatory Bowel Disease: A Randomized Trial. J. Inflamm. Res..

[77] Roifman I., Sun Y.C., Fedwick J.P., Panaccione R., Buret A.G., Liu H., Rostom A., Anderson T.J., Beck P.L. (2009). Evidence of Endothelial Dysfunction in Patients With Inflammatory Bowel Disease. Clin. Gastroenterol. Hepatol..

[78] Andreozzi M., Giugliano F.P., Strisciuglio T., Pirozzi E., Papparella A., Caprio A.M., Miele E., Strisciuglio C., Perrone Filardi P. (2019). The Role of Inflammation in the Endothelial Dysfunction in a Cohort of Pediatric Patients With Inflammatory Bowel Disease. J. Pediatr. Gastroenterol. Nutr..

[79] Triantafyllou C., Nikolaou M., Ikonomidis I., Bamias G., Kouretas D., Andreadou I., Tsoumani M., Thymis J., Papaconstantinou I. (2021). Effects of Anti-Inflammatory Treatment and Surgical Intervention on Endothelial Glycocalyx, Peripheral and Coronary Microcirculatory Function and Myocardial Deformation in Inflammatory Bowel Disease Patients: A Two-Arms Two-Stage Clinical Trial. Diagnostics.

[80] Lu Q., Shi R., Mao T., Wang Z., Sun Z., Tan X., Wang Y., Li J. (2021). Arterial Stiffness in Inflammatory Bowel Disease: An Updated Systematic Review and Meta-Analysis. Turk. J. Gastroenterol..

[81] Zanoli L., Ozturk K., Cappello M., Inserra G., Geraci G., Tuttolomondo A., Torres D., Pinto A., Duminuco A., Riguccio G. (2019). Inflammation and Aortic Pulse Wave Velocity: A Multicenter Longitudinal Study in Patients With Inflammatory Bowel Disease. J. Am. Heart Assoc..

[82] Zanoli L., Mikhailidis D.P., Bruno R.M., Abreu M.T., Danese S., Eliakim R., Gionchetti P., Katsanos K.H., Kirchgesner J., Koutroubakis I.E. (2020). Aortic Stiffening Is an Extraintestinal Manifestation of Inflammatory Bowel Disease: Review of the Literature and Expert Panel Statement. Angiology.

[83] Zivkovic P.M., Matetic A., Tadin Hadjina I., Rusic D., Vilovic M., Supe-Domic D., Borovac J.A., Mudnic I., Tonkic A., Bozic J. (2020). Serum Catestatin Levels and Arterial Stiffness Parameters Are Increased in Patients with Inflammatory Bowel Disease. J. Clin. Med..

[84] Muro P., Zhang L., Li S., Zhao Z., Jin T., Mao F., Mao Z. (2024). The Emerging Role of Oxidative Stress in Inflammatory Bowel Disease. Front. Endocrinol..

[85] Dudzińska E., Gryzinska M., Ognik K., Gil-Kulik P., Kocki J. (2018). Oxidative Stress and Effect of Treatment on the Oxidation Product Decomposition Processes in IBD. Oxidative Med. Cell. Longev..

[86] Merino De Paz N., Carrillo-Palau M., Hernández-Camba A., Abreu-González P., De Vera-González A., González-Delgado A., Martín-González C., González-Gay M.Á., Ferraz-Amaro I. (2024). Association of Serum Malondialdehyde Levels with Lipid Profile and Liver Function in Patients with Inflammatory Bowel Disease. Antioxidants.

[87] Lu Y., Li X., Liu S., Zhang Y., Zhang D. (2018). Toll-like Receptors and Inflammatory Bowel Disease. Front. Immunol..

[88] Libby P. (2021). Targeting Inflammatory Pathways in Cardiovascular Disease: The Inflammasome, Interleukin-1, Interleukin-6 and Beyond. Cells.

[89] Setyawan J., Mu F., Zichlin M.L., Billmyer E., Downes N., Yang H., Azimi N., Strand V., Yarur A. (2022). Risk of Thromboembolic Events and Associated Healthcare Costs in Patients with Inflammatory Bowel Disease. Adv. Ther..

[90] Yu S., Sun Y., Shao X., Zhou Y., Yu Y., Kuai X., Zhou C. (2022). Leaky Gut in IBD: Intestinal Barrier–Gut Microbiota Interaction. J. Microbiol. Biotechnol..

[91] Vindigni S.M., Zisman T.L., Suskind D.L., Damman C.J. (2016). The Intestinal Microbiome, Barrier Function, and Immune System in Inflammatory Bowel Disease: A Tripartite Pathophysiological Circuit with Implications for New Therapeutic Directions. Ther. Adv. Gastroenterol..

[92] Nesci A., Carnuccio C., Ruggieri V., D’Alessandro A., Di Giorgio A., Santoro L., Gasbarrini A., Santoliquido A., Ponziani F.R. (2023). Gut Microbiota and Cardiovascular Disease: Evidence on the Metabolic and Inflammatory Background of a Complex Relationship. Int. J. Mol. Sci..

[93] Wu H., Xu M., Hao H., Hill M.A., Xu C., Liu Z. (2022). Endothelial Dysfunction and Arterial Stiffness in Patients with Inflammatory Bowel Disease: A Systematic Review and Meta-Analysis. J. Clin. Med..

[94] Cibor D. (2016). Endothelial Dysfunction in Inflammatory Bowel Diseases: Pathogenesis, Assessment and Implications. World J. Gastroenterol..

[95] Xu C., Song Z., Hu L., Tong Y., Hu J., Shen H. (2024). Abnormal Platelet Parameters in Inflammatory Bowel Disease: A Systematic Review and Meta-Analysis. BMC Gastroenterol..

[96] Irving P.M., Pasi K.J., Rampton D.S. (2005). Thrombosis and Inflammatory Bowel Disease. Clin. Gastroenterol. Hepatol..

[97] Irving P.M., Macey M.G., Shah U., Webb L., Langmead L., Rampton D.S. (2004). Formation of Platelet-Leukocyte Aggregates in Inflammatory Bowel Disease. Inflamm. Bowel Dis..

[98] Bambo G.M., Shiferaw E., Melku M. (2022). A Mean Platelet Volume in Inflammatory Bowel Disease: A Systematic Review and Meta-Analysis. PLoS ONE.

[99] Kim D.S., Moon W. (2025). Coagulopathy and Platelet Abnormalities in Patients with Inflammatory Bowel Disease. Korean J. Intern. Med..

[100] Collins C.E., Cahill M.R., Newland A.C., Rampton D.S. (1994). Platelets Circulate in an Activated State in Inflammatory Bowel Disease. Gastroenterology.

[101] Andoh A., Yoshida T., Yagi Y., Bamba S., Hata K., Tsujikawa T., Kitoh K., Sasaki M., Fujiyama Y. (2006). Increased Aggregation Response of Platelets in Patients with Inflammatory Bowel Disease. J. Gastroenterol..

[102] Martinod K., Deppermann C. (2021). Immunothrombosis and Thromboinflammation in Host Defense and Disease. Platelets.

[103] Thakur M., Junho C.V.C., Bernhard S.M., Schindewolf M., Noels H., Döring Y. (2023). NETs-Induced Thrombosis Impacts on Cardiovascular and Chronic Kidney Disease. Circ. Res..

[104] Drury B., Hardisty G., Gray R.D., Ho G. (2021). Neutrophil Extracellular Traps in Inflammatory Bowel Disease: Pathogenic Mechanisms and Clinical Translation. Cell. Mol. Gastroenterol. Hepatol..

[105] Zhang L., Zheng B., Bai Y., Zhou J., Zhang X., Yang Y., Yu J., Zhao H., Ma D., Wu H. (2023). Exosomes-transferred LINC00668 Contributes to Thrombosis by Promoting NETs Formation in Inflammatory Bowel Disease. Adv. Sci..

[106] Danese S., De La Motte C., Sturm A., Vogel J.D., West G.A., Strong S.A., Katz J.A., Fiocchi C. (2003). Platelets Trigger a CD40-Dependent Inflammatory Response in the Microvasculature of Inflammatory Bowel Disease Patients. Gastroenterology.

[107] Serra G., Incani A., Serreli G., Porru L., Melis M.P., Tuberoso C.I.G., Rossin D., Biasi F., Deiana M. (2018). Olive Oil Polyphenols Reduce Oxysterols -Induced Redox Imbalance and pro-Inflammatory Response in Intestinal Cells. Redox Biol..

[108] Zou X., Zeng M., Zheng Y., Zheng A., Cui L., Cao W., Wang X., Liu J., Xu J., Feng Z. (2023). Comparative Study of Hydroxytyrosol Acetate and Hydroxytyrosol in Activating Phase II Enzymes. Antioxidants.

[109] Montoya T., Castejón M.L., Sánchez-Hidalgo M., González-Benjumea A., Fernández-Bolaños J.G., Alarcón de-la-Lastra C. (2019). Oleocanthal Modulates LPS-Induced Murine Peritoneal Macrophages Activation via Regulation of Inflammasome, Nrf-2/HO-1, and MAPKs Signaling Pathways. J. Agric. Food Chem..

[110] Montoya T., Sánchez-Hidalgo M., Castejón M.L., Rosillo M.Á., González-Benjumea A., Alarcón-de-la-Lastra C. (2021). Dietary Oleocanthal Supplementation Prevents Inflammation and Oxidative Stress in Collagen-Induced Arthritis in Mice. Antioxidants.

[111] Karagiannis T.C., Ververis K., Liang J.J., Pitsillou E., Kagarakis E.A., Yi D.T.Z., Xu V., Hung A., El-Osta A. (2024). Investigation of the Anti-Inflammatory Properties of Bioactive Compounds from Olea Europaea: In Silico Evaluation of Cyclooxygenase Enzyme Inhibition and Pharmacokinetic Profiling. Molecules.

[112] Katsa M.E., Ketselidi K., Kalliostra M., Ioannidis A., Rojas Gil A.P., Diamantakos P., Melliou E., Magiatis P., Nomikos T. (2024). Acute Antiplatelet Effects of an Oleocanthal-Rich Olive Oil in Type II Diabetic Patients: A Postprandial Study. Int. J. Mol. Sci..

[113] Zhang R., Moscona A., Myndzar K., Luttrell-Williams E., Vanegas S., Jay M.R., Calderon K., Berger J.S., Heffron S.P. (2021). More Frequent Olive Oil Intake Is Associated with Reduced Platelet Activation in Obesity. Nutr. Metab. Cardiovasc. Dis..

[114] Cohen-Mekelburg S., Van T., Wallace B., Berinstein J., Yu X., Lewis J., Hou J., Dominitz J.A., Waljee A.K. (2022). The Association Between Nonsteroidal Anti-Inflammatory Drug Use and Inflammatory Bowel Disease Exacerbations: A True Association or Residual Bias?. Am. J. Gastroenterol..

[115] Wakasugi D., Kondo S., Ferdousi F., Mizuno S., Yada A., Tominaga K., Takahashi S., Isoda H. (2024). A Rare Olive Compound Oleacein Functions as a TrkB Agonist and Mitigates Neuroinflammation Both in Vitro and in Vivo. Cell Commun. Signal..

[116] Gutiérrez-Miranda B., Gallardo I., Melliou E., Cabero I., Álvarez Y., Hernández M., Magiatis P., Hernández M., Nieto M.L. (2023). Treatment with the Olive Secoiridoid Oleacein Protects against the Intestinal Alterations Associated with EAE. Int. J. Mol. Sci..

[117] Stadnicki A., Stadnicka I. (2021). Venous and Arterial Thromboembolism in Patients with Inflammatory Bowel Diseases. World J. Gastroenterol..

[118] Valls R.-M., Farràs M., Suárez M., Fernández-Castillejo S., Fitó M., Konstantinidou V., Fuentes F., López-Miranda J., Giralt M., Covas M.-I. (2015). Effects of Functional Olive Oil Enriched with Its Own Phenolic Compounds on Endothelial Function in Hypertensive Patients. A Randomised Controlled Trial. Food Chem..

[119] Schwingshackl L., Christoph M., Hoffmann G. (2015). Effects of Olive Oil on Markers of Inflammation and Endothelial Function—A Systematic Review and Meta-Analysis. Nutrients.

[120] Derakhshandeh-Rishehri S., Kazemi A., Shim S.R., Lotfi M., Mohabati S., Nouri M., Faghih S. (2023). Effect of Olive Oil Phenols on Oxidative Stress Biomarkers: A Systematic Review and Dose–Response Meta-analysis of Randomized Clinical Trials. Food Sci. Nutr..

[121] Brata V.D., Crisan D.A., Cozma A., Gerdanovics C.-A., Popa S.L., Milaciu M.V., Orășan O.H. (2026). From Inflammation to Thrombosis: The Prothrombotic State and Cardiovascular Risk in Inflammatory Bowel Disease. Medicina.

[122] Comer-Calder H., Morad H.O.J. (2026). The Paradox of NET Involvement in the Pathogenesis of Inflammatory Bowel Disease. Inflamm. Bowel Dis..

[123] Morvaridi M., Jafarirad S., Seyedian S.S., Alavinejad P., Cheraghian B. (2020). The Effects of Extra Virgin Olive Oil and Canola Oil on Inflammatory Markers and Gastrointestinal Symptoms in Patients with Ulcerative Colitis. Eur. J. Clin. Nutr..

[124] Fitó M., Cladellas M., De La Torre R., Martí J., Alcántara M., Pujadas-Bastardes M., Marrugat J., Bruguera J., López-Sabater M.C., Vila J. (2005). Antioxidant Effect of Virgin Olive Oil in Patients with Stable Coronary Heart Disease: A Randomized, Crossover, Controlled, Clinical Trial. Atherosclerosis.

[125] Sanchez-Rodriguez E., Biel-Glesson S., Fernandez-Navarro J.R., Calleja M.A., Espejo-Calvo J.A., Gil-Extremera B., De La Torre R., Fito M., Covas M.-I., Vilchez P. (2019). Effects of Virgin Olive Oils Differing in Their Bioactive Compound Contents on Biomarkers of Oxidative Stress and Inflammation in Healthy Adults: A Randomized Double-Blind Controlled Trial. Nutrients.

[126] Estruch R., Ros E., Salas-Salvadó J., Covas M.-I., Corella D., Arós F., Gómez-Gracia E., Ruiz-Gutiérrez V., Fiol M., Lapetra J. (2018). Primary Prevention of Cardiovascular Disease with a Mediterranean Diet Supplemented with Extra-Virgin Olive Oil or Nuts. N. Engl. J. Med..

[127] Guasch-Ferré M., Hu F.B., Martínez-González M.A., Fitó M., Bulló M., Estruch R., Ros E., Corella D., Recondo J., Gómez-Gracia E. (2014). Olive Oil Intake and Risk of Cardiovascular Disease and Mortality in the PREDIMED Study. BMC Med..

[128] Urpi-Sarda M., Casas R., Sacanella E., Corella D., Andrés-Lacueva C., Llorach R., Garrabou G., Cardellach F., Sala-Vila A., Ros E. (2021). The 3-Year Effect of the Mediterranean Diet Intervention on Inflammatory Biomarkers Related to Cardiovascular Disease. Biomedicines.

[129] Khandouzi N., Zahedmehr A., Nasrollahzadeh J. (2021). Effect of Polyphenol-Rich Extra-Virgin Olive Oil on Lipid Profile and Inflammatory Biomarkers in Patients Undergoing Coronary Angiography: A Randomised, Controlled, Clinical Trial. Int. J. Food Sci. Nutr..

[130] Cariello M., Contursi A., Gadaleta R.M., Piccinin E., De Santis S., Piglionica M., Spaziante A.F., Sabbà C., Villani G., Moschetta A. (2020). Extra-Virgin Olive Oil from Apulian Cultivars and Intestinal Inflammation. Nutrients.

[131] Voltes A., Bermúdez A., Rodríguez-Gutiérrez G., Reyes M.L., Olano C., Fernández-Bolaños J., Portilla F.D.L. (2020). Anti-Inflammatory Local Effect of Hydroxytyrosol Combined with Pectin-Alginate and Olive Oil on Trinitrobenzene Sulfonic Acid-Induced Colitis in Wistar Rats. J. Investig. Surg..

[132] Sarapis K., Thomas C.J., Hoskin J., George E.S., Marx W., Mayr H.L., Kennedy G., Pipingas A., Willcox J.C., Prendergast L.A. (2020). The Effect of High Polyphenol Extra Virgin Olive Oil on Blood Pressure and Arterial Stiffness in Healthy Australian Adults: A Randomized, Controlled, Cross-Over Study. Nutrients.

